# A Short Fully Covered Self-Expandable Metal Stent for Management of Benign Biliary Stricture Not Caused by Living-Donor Liver Transplantation

**DOI:** 10.3390/jcm13051186

**Published:** 2024-02-20

**Authors:** See-Young Lee, Sung-Ill Jang, Moon-Jae Chung, Jae-Hee Cho, Min-Young Do, Hye-Sun Lee, Juyeon Yang, Dong-Ki Lee

**Affiliations:** 1 Department of Internal Medicine, Institute of Gastroenterology, Yonsei University College of Medicine, Seoul 03722, Republic of Korea; seeyoung87@yuhs.ac (S.-Y.L.); aerojsi@yuhs.ac (S.-I.J.); mjchung@yuhs.ac (M.-J.C.); jhcho9328@yuhs.ac (J.-H.C.); dmy24@yuhs.ac (M.-Y.D.); 2 Biostatistics Collaboration Unit, Yonsei University College of Medicine, Seoul 03722, Republic of Korea; hslee1@yuhs.ac (H.-S.L.); ju1003yeon@yuhs.ac (J.Y.)

**Keywords:** benign biliary stricture, endoscopic retrograde cholangiopancreatography, fully covered self-expanding metal stent

## Abstract

**Background**: This study evaluated the effectiveness of short fully covered self-expanding metal stents (FCSEMS) with an anti-migration design in treating benign biliary strictures (BBS) not related to living donor liver transplantation (LDLT). **Methods**: A retrospective analysis was conducted on 75 patients who underwent FCSEMS insertion for BBS management. Stents were initially kept for 3 months and exchanged every 3 months until stricture resolution. Adverse events and stricture recurrence after FCSEMS removal were assessed during follow-up. **Results**: The study outcomes were technical success, stenosis resolution, and treatment failure. Technical success was 100%, with stricture resolution in 99% of patients. The mean onset time of BBS post-surgery was 4.4 years, with an average stent indwelling period of 5.5 months. Stricture recurrence occurred in 20% of patients, mostly approximately 18.8 months after stent removal. Early cholangitis and stent migration were noted in 3% and 4% of patients, respectively. **Conclusions**: This study concludes that short FCSEMS demonstrate high efficacy in the treatment of non-LDLT-related BBS, with a low incidence of interventions and complications. Although this is a single-center, retrospective study with a limited sample size, the findings provide preliminary evidence supporting the use of short FCSEMS as a primary treatment modality for BBS. To substantiate these findings, further research involving multicenter studies is recommended to provide additional validation and a broader perspective.

## 1. Introduction

Benign biliary strictures (BBSs) are clinically significant because they can cause jaundice, chronic cholestasis, and cholangitis. The course of disease in patients with BBS is initially asymptomatic with biochemical changes in liver enzymes, making diagnosis difficult. As the disease progresses, pronounced jaundice may develop, secondary to cholangitis. These complications not only cause considerable discomfort to the patient but, if left untreated, can lead to biliary cirrhosis or death [1,2].

Advances in medical technology and surgical techniques have expanded the indications for liver and gallbladder interventions, resulting in the emergence of BBS as a notable postoperative complication [3]. Indeed, surgery-related BBS accounts for a substantial proportion of all BBS cases. BBS is mainly seen as a consequence of diseases such as cirrhosis or procedures such as bile duct surgery and liver transplantation, with reported incidences of 0.5% after laparoscopic cholecystectomy [4], 15% to 20% after deceased-donor liver transplantation (DDLT), and 19% to 40% after living-donor liver transplantation (LDLT) [5]. Furthermore, the increasing incidence of primary sclerosing cholangitis and IgG4 cholangiopathy emphasizes the importance of BBS other than biliary strictures at the site of surgical anastomosis [3].

The optimal treatment for BBS has not yet been clearly identified, but nonsurgical approaches that maintain the patient’s quality of life are increasingly favored [6]. Endoscopic management of BBS is less invasive and causes less patient morbidity than surgical or percutaneous treatments [7]. Endoscopic intervention stands out as safe, effective, and amenable to repetition. There are two main endoscopic treatments for BBS: multiple plastic stents and fully covered self-expanding metal stents (FCSEMSs). The advantages of multiple plastic stents are their low cost and few complications, such as biliary tract infection and migration, and the disadvantages are the need for repeat procedures and a high BBS recurrence rate. The advantages of FCSEMSs are the need for fewer repeat procedures and a relatively low BBS recurrence rate, but the disadvantages are their high cost and possible complications of biliary tract infection and migration [3,7,8]. Therefore, comparative studies between the two stent types continue to be conducted, and recent evidence emphasizes the benefits of using FCSEMSs for the treatment of BBS [9,10,11].

Short FCSEMSs with an anti-migration design are gaining traction in the clinic, as they improve on the disadvantages of FCSEMSs. Short FCSEMSs are structurally modified by reducing the length of the stent and creating an anti-migration waist to reduce the complications of biliary tract inflammation and migration with FCSEMS use [12,13]. Previous studies first described the effectiveness of short FCSEMSs with an anti-migration design, particularly for BBS occurring after LDLT, as LDLT is the leading cause of BBS [14,15,16]. Given the complex nature of the donor–recipient anastomosis in LDLT compared with other surgical techniques, the management of BBS after LDLT is important. However, as mentioned earlier, a significant number of BBSs also occur due to causes other than LDLT, and addressing them is equally important and should not be underestimated. To date, there is a lack of research analyzing cases treated with short FCSEMSs for BBS not caused by LDLT.

Therefore, in this study, we aimed to evaluate the effectiveness of a short FCSEMS with an anti-migration design in treating BBS caused by various biliary surgical procedures other than LDLT and BBS occurring unrelated to surgery.

## 2. Materials and Methods

### 2.1. Patients

We conducted a retrospective analysis of data from 75 patients who underwent endoscopic placement of a short FCSEMS for BBS treatment between January 2015 and July 2022 at Gangnam Severance Hospital and Severance Hospital, Republic of Korea. The indication for ERCP was established in patients with BBS who had biliary obstruction confirmed by laboratory or radiological findings. If cholangiography confirmed biliary stricture, placement of FCSEMS was implemented regardless of laboratory findings. Inclusion criteria were (i) patients aged 18 years or older and (ii) patients with BBS developed after biliary surgery or without biliary surgery. Criteria for exclusion were (i) anastomotic stricture after LDLT, (ii) contraindications to endoscopic interventions, and (iii) malignant strictures, as determined by pathological examination or clinical progression. This study received approval from the Institutional Review Board of Gangnam Severance Hospital, Republic of Korea (IRB number 3-2015-0301). All participants enrolled in the study provided their written informed consent.

### 2.2. Stents

**Figure 1:**
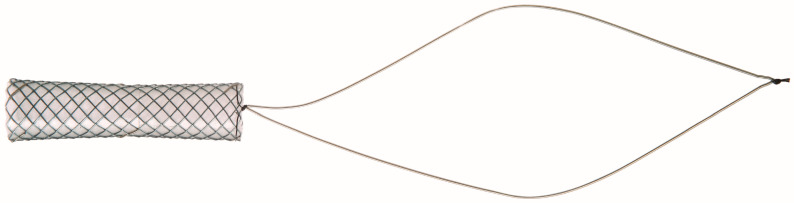
An example of a short FCSEMS (Niti-S KAFFES Biliary Stent). This biliary stent is engineered for complete insertion within the biliary tract and features a retrieval string that extends to the duodenum, facilitating its extraction from the common bile duct. Its distinctive design incorporates a ‘waist’ that gradually tapers at the midpoint, effectively preventing migration. Additionally, the stent is equipped with radiopaque markers: three are positioned at each end and two in the middle, enabling precise placement of the metal stent at the center of the stricture.

The short FCSEMS employed in this research (Niti-S KAFFES™ Biliary Stent; Taewoong Medical Co., Ltd., Gimpo-si, Republic of Korea) is characterized by three distinct features: (i) a unique design in which the stent maintains a consistent diameter at both extremities but tapers toward the center, which aids in preventing stent migration; (ii) a shorter length relative to conventional stents, ensuring optimal coverage of the stenotic region while limiting contact with the healthy bile duct, thereby minimizing potential damage to the uninvolved biliary tissue; and (iii) the inclusion of a long retrieval string marked with platinum radiopaque indicators affixed to the stent’s distal end, designed to enhance stent retrieval using standard endoscopic biopsy forceps. In this study, we used stents with lengths of 4, 5, 6, and 8 cm and diameters of 6, 8, and 10 mm based on the characteristics of the biliary stricture in question (Figure 1).

### 2.3. Interventions

**Figure 2:**
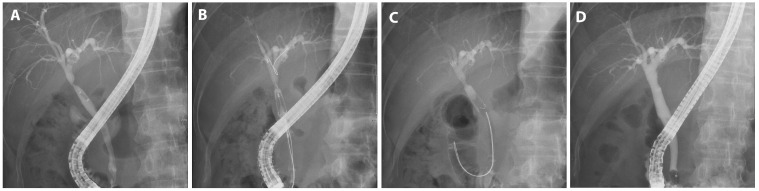
FCSEMS treatment of biliary anastomotic stricture after cholecystectomy. A case of biliary anastomotic stricture diagnosed after cholecystectomy and treated with FCSEMSs is presented. (**A**) A biliary anastomotic stricture that had developed after surgery was observed in the common bile duct on cholangiography. (**B**) The stent was positioned before deployment such that the central radiopaque marker was aligned with the stenosis. (**C**) An FCSEMS (6 mm in diameter, 4 cm in length; KAFFES) was deployed at the site of biliary anastomotic stricture. (**D**) After 9 months of FCSEMS use, including two replacements, the stent was removed, and the procedure was terminated after confirming that the biliary anastomotic stricture had resolved.

Each endoscopic retrograde cholangiopancreatograph (ERCP) was performed by one of five experienced endoscopists, each with more than five years of experience and more than 1000 successful ERCPs. Throughout the intervention, patients were given a sedative regimen comprising propofol, midazolam, and pethidine while being continuously monitored either by an anesthesiologist or the performing endoscopist. The procedure was performed using duodenoscopes (TJF-260 and TJF-290V; Olympus Medical Systems Corp., Tokyo, Japan) equipped with a 4.2 mm instrument channel, ensuring precise diagnosis and therapeutic intervention. After bile duct cannulation, a guide wire was inserted through the stricture. The stenosis length and the diameter of the proximally dilated intrahepatic duct, as discerned on the cholangiogram, informed the choice of stent dimension, ensuring comprehensive coverage of the stenotic segment (Figure 2A). The placement of the stent was carefully adjusted to ensure the alignment of its central radiopaque marker with the site of the stenosis (Figure 2B). In cases of severe stenosis that hindered catheter insertion, either a balloon dilator (6 mm or 8 mm, Hurricane RX; Boston Scientific, Marlborough, MA, USA) or a bougie dilator (Soehendra Biliary Dilation Catheter; Cook Medical, Bloomington, IN, USA) was used as a preliminary intervention. The stent body was placed inside the bile duct, and the retrieval string was pulled out of the papilla and positioned in the duodenum. Each short FCSEMS was retained for 3 months before being meticulously extracted using endoscopic forceps. Then, cholangiography was undertaken to verify rectification of the BBS (Figure 2D). To ward off potential infections, prophylactic antibiotics were judiciously administered both pre- and post-procedure.

**Figure 3:**
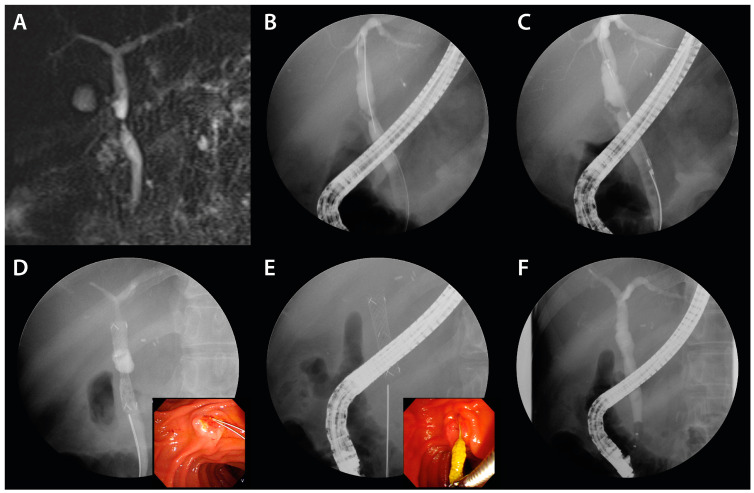
FCSEMS treatment of biliary anastomotic stricture after DDLT. A case of biliary anastomotic stricture that developed after DDLT for alcoholic cirrhosis and treated with FCSEMSs is presented. (**A**) A magnetic resonance cholangiopancreatograph was performed to visualize the shape and length of the stricture. A common bile duct stricture is shown. (**B**) A common bile duct stricture was observed on cholangiogram. (**C**) An FCSEMS (8 mm in diameter, 4 cm in length; KAFFES) stent was deployed so that the central radiopaque marker was centered in the stenosis. (**D**) The FCSEMS is shown at the stricture site, and the retrieval string is located at the duodenum (color figure). (**E**) After stent indwelling for 3 months, the FCSEMS was found to be completely self-inflating, and the FCSEMS is removed by grasping the retrieval string using grasping forceps (color panel). (**F**) After three months of FCSEMS, including one replacement, the stent was removed and the biliary anastomotic stricture resolved.

In instances where the stenosis persisted, the FCSEMS was reinserted. The stent was removed by pulling the retrieval string, which was visible in the duodenum, using endoscopic graspers (Figure 3E). After the procedure, patients were scheduled at 3 month intervals based on previous studies and considering the risk of stent occlusion and cholangitis [15], and were followed up every 1 month after stent placement to monitor for signs of stent occlusion. Cycles of FCSEMS deployment via ERCP were performed for up to 12 months or until complete resolution of BBS. The FCSEMS was positioned using the rendezvous technique when biliary cannulation was challenging or when a patient had previously undergone percutaneous transhepatic biliary drainage (PTBD). Moreover, in cases of BBS proximate to the hilar region, a precautionary measure was employed: a 7-Fr plastic stent (Zimmon, double pigtail type; Cook Medical) was pre-emptively placed prior to the FCSEMS insertion, especially in scenarios indicating potential blockage of adjacent biliary branches by the FCSEMS. In the subset of patients exhibiting multiple BBS presentations, as many as two FCSEMS were judiciously deployed to address each stenotic site.

### 2.4. Follow-Up and Outcomes

Following discharge, patients who experienced successful stenting were consistently monitored during outpatient visits to track clinical symptoms, detect any laboratory abnormalities, and observe potential adverse events. A month after stent insertion, abdominal radiograph images were obtained to see if any stent migration had transpired, and serological tests were performed to identify any onset of cholangitis, the most frequently observed complication. For patients with resolved BBS who had eventual stent removal, outpatient follow-up was performed every 3 months with a focus on identifying any laboratory findings suggestive of recurrence or worsening of clinical symptoms. If re-stenosis was suspected due to worsening clinical symptoms or deviations in serological findings, a comprehensive evaluation was initiated, including computed tomography, magnetic resonance cholangiography, and a diisopropyl iminodiacetic acid scan. If re-stenosis was evident by these evaluations, cholangiography with ERCP was performed for a final diagnosis of recurrent BBS, followed by placement of another FCSEMS.

Technical success was defined as the accurate placement of the stent at the stenosis site, the successful administration of the contrast agent, and the unobstructed flow of the contrast agent through the stent. Treatment failure was defined as the occurrence of either major or minor stent-related complications post-ERCP, such as acute cholangitis or stent migration. An early complication was denoted if such adverse events manifested up to 1 month following the procedure, whereas complications presenting after 1 month were categorized as late complications. Stenosis resolution was defined based on cholangiogram findings of no narrowing of the stenosis at the time of stent removal, the ability of the dilated balloon catheter to pass through the stenosis without resistance, the unimpeded flow to the distal portion of a contrast agent administered proximal to the stenosis, and no evidence of bile duct obstruction observed within 3 days of FCSEMS removal or during subsequent follow-up. If these stenosis resolution criteria were met, the stent was removed. If these criteria were not met, the stent was replaced. After a successful BBS resolution, stenosis recurrence was defined as the event wherein imaging evaluation during outpatient follow-up revealed an obstruction localized at the biliary anastomosis site that required further intervention.

### 2.5. Statistical Analysis

Continuous variables adhering to a normal distribution are described using the mean and standard deviation (SD), while those deviating from normal distribution are presented as the median along with the interquartile range (IQR). To analyze differences in continuous variables, statistical methods such as the Mann–Whitney U, Kruskal–Wallis, and Wilcoxon rank sum tests were utilized. Frequencies and percentages were used to express categorical variables, with the chi-square or Fisher’s exact test applied as suitable for their assessment. Success and recurrence rates among outcomes were treated as binary data. The Kaplan–Meier approach was employed for analyzing stenosis recurrence and stricture resolution. Statistical analyses were conducted using SPSS version 23.0 (IBM Corp., Armonk, NY, USA) and R software version 4.1.0 (R Foundation for Statistical Computing, Vienna, Austria), with a *p*-value below 0.05 being considered as indicative of statistical significance.

## 3. Results

We treated 75 patients with BBS using short FCSEMSs. The mean age of the participants was 58.3 years; 55 were male and 20 were female. The most common surgery performed on these patients was cholecystectomy, accounting for 41.3% (31 of 75) of patients. This was followed by DDLT, which accounted for 30.7% (23 of 75) of patients. Liver resection was performed in 20.0% (15 of 75) of patients. The remaining 8.0% (6 of 75) of patients were diagnosed with benign strictures. In this study, we presented procedural images of representative cases by cause. A patient who underwent a therapeutic ERCP procedure for biliary stricture following cholecystectomy, a major cause of BBS independent of LDLT, experienced recovery after 9 months of FCSEMS treatment, including two replacement procedures (Figure 2).

A case manifesting common bile duct (CBD) stricture subsequent to DDLT found resolution after a three-month FCSEMS application (Figure 3).

**Figure 4:**
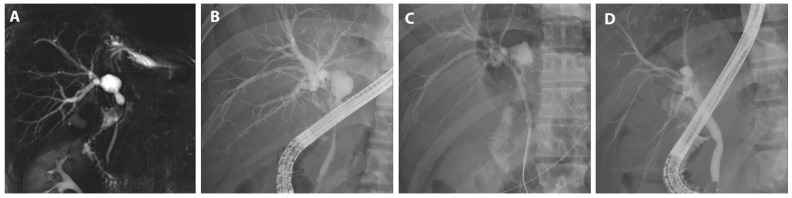
FCSEMS treatment of biliary anastomotic stricture after liver resection. A case diagnosed with biliary anastomotic stricture after left hemihepatectomy for liver donation and treated with FCSEMSs is presented. (**A**) Magnetic resonance cholangiopancreatography shows segmental narrowing at the hilar and common hepatic duct with mild central intrahepatic bile duct dilatation, and a 2 cm-sized biloma was observed at the resection margin. (**B**) A hilar stricture was observed on cholangiogram. (**C**) An FCSEMS (6 mm in diameter, 5 cm in length; KAFFES) was deployed at the site of biliary anastomotic stenosis, with a plastic stent placed prophylactically, as expansion of the FCSEMS could narrow the adjacent branches. (**D**) After the FCSEMS was in use for 6 months, including one replacement, the stent was removed, and the procedure was terminated after confirming that the biliary anastomotic stricture was resolved.

A patient subjected to hemi-hepatectomy developed a hilar stricture that was treated using FCSEMS; additionally, a plastic stent was prophylactically deployed in another bile duct to prevent cholangitis (Figure 4).

**Figure 5:**
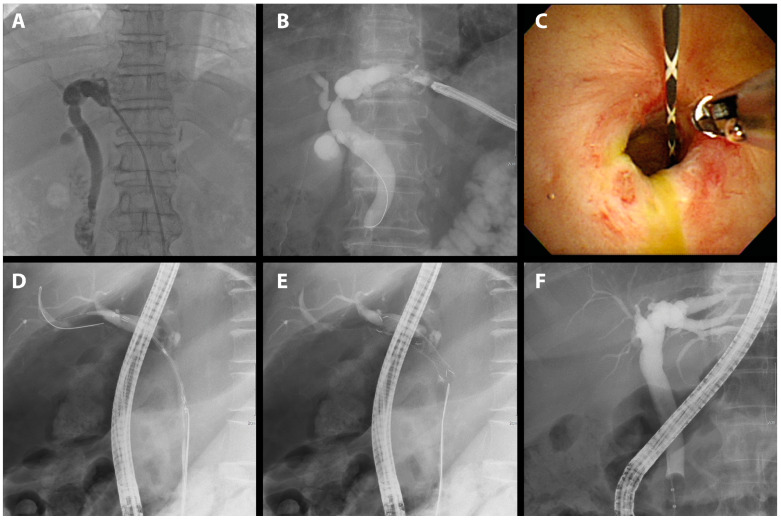
FCSEMS treatment of BBS. A case of BBS diagnosed by histological findings and treated with FCSEMSs is presented. (**A**) Cholangiography with PTBD confirmed left biliary stricture. (**B**) Percutaneous transhepatic cholangioscopy was used for histological examination. (**C**) Histological examination was performed on the area of biliary stricture. (**D**) An FCSEMS (10 mm in diameter, 4 cm in length; KAFFES) was inserted into the BBS. (**E**) The FCSEMS was successfully deployed. (**F**) After 12 months of FCSEMS use, including three replacements, the stent was removed, and the procedure was terminated after confirming that the BBS had resolved.

Despite the potential of non-surgical BBS emanating from diverse etiologies including primary sclerosing cholangitis and IgG4-related cholangiopathy, their representation in our study was scant. Hence, we consolidated non-surgical BBS cases into a singular category. For the patient presented in this study, only benign fibrotic tissue was confirmed histologically, and there were no IgG4 or other pathological findings, so the patient is being followed up on an outpatient basis after treatment of the stricture (Figure 5).

In terms of the number of strictures per patient, the majority, 55 (73.3%) had a single stricture and 20 (26.7%) had multiple strictures. On average, it took 52.7 months for a stenosis to be diagnosed after surgery. Regarding previous procedures, the largest group of patients, 25 (33.33%), underwent ERCP alone, 11 (14.67%) underwent PTBD alone, 19 (25.33%) underwent both procedures (ERCP + PTBD), and 20 (26.67%) underwent neither procedure (Table 1).

Most patients (67 (89.3%)) had an FCSEMS inserted by endoscopic methods alone, while 8 (10.7%) had an FCSEMS inserted using the rendezvous method. The most common stent length used was 4 cm (44 patients), followed by 6 cm (26 patients). The most common stent diameter used was 8 mm (40 patients), followed by 6 mm (26 patients). Twenty-seven (36.0%) patients underwent balloon dilation prior to FCSEMS insertion, and 22 (29.3%) patients had an additional plastic stent inserted along with the FCSEMS. The mean total indwelling period (i.e., the total time the stent was in the patient) was 5.5 months, and the mean indwelling period per FCSEMS was 3.1 months. Twenty-four (32.0%) patients required a stent exchange during the study period. The number of stent exchanges varied, with 44 patients requiring no exchanges, 21 requiring one exchange, 7 requiring two exchanges, and 3 requiring three exchanges. At stent removal, sludge was observed in 25 (33.3%) patients, and common or intrahepatic bile duct stones were found in 7 (9.3%) patients (Table 2).

Every procedure conducted on the cohort achieved technical success, reflected in a 100.0% (75 of 75 patients) technical success rate. By the time of the final FCSEMS removal, an encouraging 98.7% (74 of 75 patients) had a resolution of their stricture, underscoring the efficacy of the stent placement. After the removal of the stent, the patients were systematically followed up for a mean period of 47.5 months. In terms of recurrence, 20.0% (15 of 75 patients) experienced a return of the stricture after stent removal. On further examination of the duration dynamics, it was observed that the stricture typically recurred approximately 18.8 months after stent removal. Conversely, the mean duration that patients remained free from the recurrence of stricture after the initial procedure was 40.9 months. Post-procedure complications within 1 month of the procedure were cholangitis in two (2.67%) patients. After 1 month post-procedure, asymptomatic stent migration was reported in three (4.00%) patients and cholangitis in one (1.33%) patient. Various treatments were administered to address the cases of recurrence. Most patients (11 of 15) underwent a second insertion of the FCSEMS, for which the KAFFES stent was specifically used. A smaller subset, two patients, required the placement of an endoscopic retrograde biliary drainage plastic stent. Only one patient required a PTBD intervention. One other case from the cohort received a hepatectomy. The mean total bilirubin levels showed a decrease from a baseline of 4.0 mg/dL to a value on day 1 post-procedure of 3.4 mg/dL, and further decreased to 1.5 mg/dL by day 28 (Table 3).

**Figure 6:**
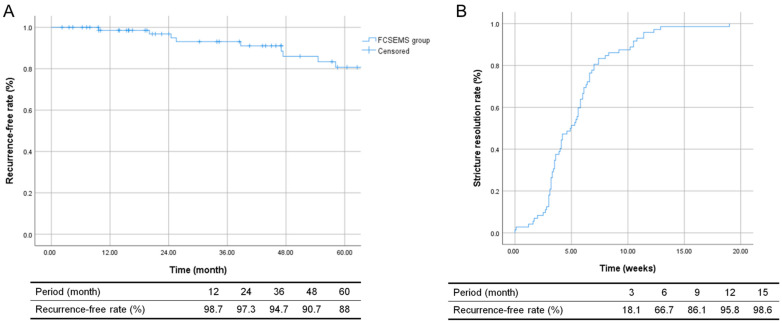
Kaplan–Meier curves on stricture resolution and recurrence. (**A**) The cumulative recurrence-free rates for a short FCSEMS use were 98.5% (95% CI: 0.96–1.00) at one year and 80.7% (95% CI: 0.70–0.93) at five years. (**B**) The stricture resolution rates for a short FCSEMS were 66.7% (95% CI: 0.53–0.79) at six months and 95.8% (95% CI: 0.93–0.99) at one year.

The cumulative recurrence-free rates for a short FCSEMS use were 98.5% (95% CI: 0.96–1.00) at one year and 80.7% (95% CI: 0.70–0.93) at five years, which seemed to be associated with efficacy in the treatment of BBS. The rates of stenosis resolution were 66.7% (95% CI: 0.53–0.79) at six months and 95.8% (95% CI: 0.93–0.99) at one year following the placement of the short FCSEMS (Figure 6).

## 4. Discussion

In this study, we sought to determine the efficacy of the short FCSEMS in a variety of BBSs other than LDLT; we found that the short FCSEMS with an anti-migration design had as high a stricture-resolution rate in treating other BBSs as in LDLT. In contrast to LDLT, where substantial data is available, there is limited information on biliary strictures following cholecystectomy, DDLT, and other liver resection surgeries. This is attributed to the anastomosis site in LDLT being more peripheral and often smaller and more complex than in other surgeries [17]. Consequently, our study focused on biliary strictures occurring post-cholecystectomy, DDLT, and other surgeries, where data are relatively scarce. It is known that common postoperative biliary strictures, apart from LDLT, typically occur in the mid extrahepatic duct, usually more than 1 cm from the hilum [2]. The findings of this study thus offer a valuable clinical resource for treating BBS arising in the mid extrahepatic duct.

The common endoscopic treatment approach for these various BBSs has been the multiple plastic stent approach, which uses a balloon catheter to dilate the stenosis, followed by insertion of multiple parallel plastic stents [6]. This requires many ERCP sessions (three to four on average) to sufficiently dilate the bile duct, deploy the stent, progressively increase the stent size, and ultimately extract all of the stent [7,8]. While this multiple plastic stent approach has a high stricture-resolution rate, it has the disadvantage of requiring multiple treatment sessions. Placement of a single FCSEMS has a radial dilation effect on stenosis similar to that of at least three plastic stents placed side by side, and preliminary investigations, including small clinical trials, have shown that placement of an FCSEMS in patients with benign stenosis has similar efficacy to plastic stent treatment [16,18,19].

Recent evidence suggests using an FCSEMS for the treatment of BBS because of its advantages: superior technical and clinical success rates, simplified insertion, and reduced need for endoscopic intervention to resolve strictures [20,21,22,23]. However, its disadvantages, such as cholangitis and migration, are still a weakness of FCSEMS use and may have a more costly impact. Therefore, the short FCSEMS with anti-migration design used in this study, which compensates for the disadvantages of the conventional FCSEMS, seems to be worthy of prioritized consideration in treating various BBS types. There are no data yet on complications of short FCSEMS use for each condition for various BBSs other than LDLT. Comprehensive BBS studies, including those caused by LDLT, provide migration complication rates of 10% to 16% for conventional FCSEMSs, but the migration complication rate in this study was 4%, demonstrating the effectiveness of the waist shape that narrows the center of the stent to prevent migration [24,25,26,27]. Additionally, the presence of radiopaque markers in the center and at both ends of the stent facilitates proper positioning [15]. Of course, the recurrence rate in this study was high, at 20.0%, but the final clinical success rate after secondary short FCSEMS implantation was 73.3%. This suggests that a significant majority of the recurrent BBSs were effectively addressed after a subsequent short FCSEMS procedure.

In this study, patients exhibited BBS development at a mean duration of 52.7 months after surgery. However, a considerable SD of 69 months underscores the heterogeneity in the timeframes across different causes of BBS. This observed variance might be attributed to the limited number of patients grouped under each specific cause. Moving forward, a more nuanced analysis with a larger patient cohort, segregated by the distinct causes of BBS, would be instrumental in refining our understanding. The observed total indwelling period in our study was 5.5 months, notably shorter than the 12 month period associated with multiple plastic stent methods [2,3]. Furthermore, it is of particular significance that 44 (58.7%) patients achieved BBS resolution following just a single short FCSEMS procedure. This underscores the relatively high efficacy achieved in a condensed timeframe. Furthermore, extended stenting durations can predispose patients to complications arising from biliary tract infection, notably due to stent occlusion. Based on our results, we recommend a short FCSEMS total indwelling period of 5.5 months or less. Definitive determination of the ideal stent retention duration requires further prospective investigations, and after stenting, it is necessary to periodically check the patient’s clinical condition to remove the stent at the optimal time.

One of the most important concerns when using an FCSEMS to treat BBS is the potential occlusion of lateral branching ducts [9]. Inserting an FCSEMS into a biliary stricture carries the risk of side-branch duct occlusion or bile stasis. In the case of proximal BBS, a plastic stent should be inserted in each duct, as there may be multiple strictures surrounding the BBS. However, it is not feasible to insert many stents in one duct. To prevent obstruction in adjacent ducts, we strategically positioned a plastic stent within the bile duct collateral prior to the introduction of the FCSEMS. We also used two FCSEMSs if there were multiple BBSs. In our study, cholangitis was observed in 4.0% of cases, but the cholangitis was typically resolved through stent removal or exchange. Additionally, at a mean indwelling period of 3.1 months per FCSEMS, sludge was identified in 33.3% of cases and CBD or IHD stones in 9.3% of cases at stent removal. This can be attributed to cholestasis. To counteract the formation of stones or sludge, it is advisable to use hydrophilic membranes or make other modifications to the stent to improve bile flow.

Although cholecystectomy typically has the lowest incidence of BBS compared to other surgeries, cholecystectomy accounted for the highest percentage of BBS cases in this study [4]. This discrepancy may be due to the disproportionate number of cholecystectomies performed at our institution compared to other surgeries. Approximately 1500 cholecystectomies were performed at our institution in a single year. When the incidence of BBS was calculated against this volume of surgeries, it was found to be approximately 0.2%, which is similar to or lower than existing data. This suggests that the higher incidence of BBS after cholecystectomy observed in this study is likely due to the increased frequency of surgery rather than an increased risk inherent in the surgery itself.

The limitations of this study include its retrospective design, enrollment of only patients with BBS, and alternating use of additional plastic stenting and balloon dilation in some patients. In addition, it was a single-center study with a small number of patients, which should be validated in a larger number of patients in future multicenter studies. This study enrolled only patients with BBS treated with FCSEMSs at our institution, so it was not possible to conduct a comparative study of the efficacy of FCSEMSs for other stricture types. Subsequent comparative studies evaluating FCSEMS use compared with other modes of treatment are needed.

## 5. Conclusions

The use of short FCSEMSs was associated with high stricture-resolution rates in patients with BBS not caused by LDLT. The use of short FCSEMSs may be useful in the primary treatment of BBS and strictures that occur after biliary surgical anastomosis. Future multicenter studies with larger patient cohorts are needed to further substantiate these observations, as well as prospective comparative studies contrasting this approach with conventional guideline-recommended treatment using multiple plastic stents.

## Figures and Tables

**Table 1:** Basic patient characteristics (N = 75).

Characteristic	Data Value
Age (years), mean ± SD	58.3 ± 14.06
Male:Female, n	55:20
Type of operation, n (%)	
Cholecystectomy	31 (41.33)
DDLT	23 (30.67)
Liver resection	15 (20.00)
No operation (benign stricture)	6 (8.00)
No. of strictures	
Single: Multiple	55:20
Duration between operation and diagnosis of stricture (mo), mean ± SD	52.74 ± 69.39
Previous procedures, n (%)	
ERCP only	25 (33.33)
PTBD only	11 (14.67)
ERCP + PTBD	19 (25.33)
None	20 (26.67)

DDLT, deceased donor liver transplantation; ERCP, endoscopic retrograde cholangiopancreatography; PTBD, percutaneous transhepatic biliary drainage; SD, standard deviation.

**Table 2:** Procedure-related characteristics and outcomes (N = 75).

Characteristic or Outcome	Data Value
Method of FCSEMS insertion, n (%)	
Endoscopic method only	67 (89.30)
Rendezvous method	8 (10.70)
FCSEMS stent, n	
Length (cm): 4/5/6/8	44/4/26/1
Diameter (mm): 6/8/10	26/40/9
Procedures combined with FCSEMS placement, n (%)	
Balloon dilation before stent insertion	27 (36.00)
Insertion of an additional plastic stent	22 (29.33)
Indwelling period and exchange time	
Total indwelling period (mo), mean ± SD	5.48 ± 3.71
Indwelling period per FCSEMS (mo), mean ± SD	3.07 ± 1.30
Patients who required stent exchange, n (%)	24 (32.00)
Stent exchange, n: 0/1/2/3	44/21/7/3
Stones or sludge evident at the time of stent removal, n (%)	
Sludge	25 (33.33)
CBD or IHD stone	7 (9.33)

CBD, common bile duct; FCSEMS, fully covered self-expandable metallic stent; IHD, intrahepatic bile duct; SD, standard deviation.

**Table 3:** Outcomes of fully covered metal stent placement for BBS (N = 75).

Outcome	Data Value
Technical success rate, n (%)	75 (100.00)
Stricture resolution at final FCSEMS removal, n (%)	74 (98.67)
Overall follow-up period after stent removal (mo), mean ± SD	47.45 ± 30.03
Stricture recurrence, n (%)	15 (20.00)
Duration between stent removal and recurrence (mo), mean ± SD	18.75 ± 15.49
Recurrence-free duration (mo), mean ± SD	40.88 ± 31.30
Complications, n (%)	
Early complications (≤1 mo)	
Cholangitis	2 (2.67%)
Late complications (>1 mo)	
Asymptomatic stent migration	3 (4.00)
Cholangitis	1 (1.33)
Treatment after recurrence, n (%)	15 (20.00)
Second FCSEMS ^†^ insertion	11 (14.70)
ERBD (plastic stent) insertion	2 (2.70)
PTBD insertion	1 (1.30)
Hepatectomy	1 (1.30)
Total bilirubin (mg/dL), mean ± SD	
Baseline	4.0 ± 2.39
Day 1	3.4 ± 1.97
Day 28	1.5 ± 1.22

BBS, benign biliary stricture; ERBD, endoscopic retrograde biliary drainage; FCSEMS, fully covered self-expandable metallic stent; PTBD, percutaneous transhepatic biliary drainage; SD, standard deviation. ^†^ The FCSEMS was the KAFFES stent.

## Data Availability

The data used in this study are available in this article.
